# Lymphocyte Subtypes in Local Inflammatory Response: A Comparative Analysis of Simple and Complicated Appendicitis

**DOI:** 10.3390/medicina61122161

**Published:** 2025-12-04

**Authors:** Maja Zecevic, Dragoljub Zivanovic, Nikola Zivkovic, Aleksandra Velickov, Vesna Marjanovic, Zoran Marjanovic, Sanja Milenkovic, Aleksandar Sretenovic

**Affiliations:** 1 Clinic for Pediatric Surgery, Orthopedics and Traumatology, University Clinical Center Nis, 18000 Nis, Serbia; 2 Department of Surgery, Anesthesiology and Reanimatology, Faculty of Medicine, University of Nis, 18000 Nis, Serbia; 3 Department of Pathology and Pathological Anatomy, Faculty of Medicine, University of Nis, 18000 Nis, Serbia; 4 Department of histology and Embryology, Faculty of Medicine, University of Nis, 18000 Nis, Serbia; 5 Clinic for Anesthesiology, Intensive Therapy and Reanimatology, University Clinical Center Nis, 18000 Nis, Serbia; 6 Department of Pathology, Clinical and Hospital Center Zemun, 11080 Zemun, Serbia; 7 University Children’s Hospital Belgrade, 11000 Belgrade, Serbia; 8 Department of Surgery, Faculty of Medicine, University of Belgrade, 11000 Belgrade, Serbia

**Keywords:** children, appendicitis, lymphocytes, CD8, CD19, CD20

## Abstract

*Background and Objectives:* The immunological function of the appendix, as part of the gut-associated lymphoid tissue, and its immunological importance in developing and maintaining the intestinal immune system are not yet clearly understood. Therefore, we sought some answers by analyzing the lymphocyte population in appendiceal specimens and inflammatory markers from peripheral blood. *Materials and Methods:* After histopathological examination of appendiceal specimens, immunohistochemical analysis of lymphocyte subtypes CD8, CD19, and CD20 in appendiceal specimens was performed, and a panel of commercially available mouse monoclonal antibodies was used to detect immunopositivity. hematological and biochemical parameters were analyzed from peripheral blood samples, and results were correlated with immunohistochemical findings. *Results:* The study included 100 patients: n = 20 in the control group, where the appendix was removed during some other abdominal surgical procedure, n = 35 patients with simple appendicitis in Group A, and n = 45 with complicated appendicitis in Group B. There were 65.0% male patients, and the mean age at surgery was 10.8 ± 4.0 years. CD19 B-lymphocytes were significantly more present in the appendiceal specimens of patients with complicated appendicitis (*p* = 0.014 and *p* = 0.007), and CD8 T-lymphocytes in patients with simple appendicitis (*p* < 0.001). Also, there were significantly fewer CD8 T-lymphocytes in patients with complications (*p* = 0.024). *Conclusions:* The number of CD8 T-lymphocytes is increased in simple appendicitis and is considerably lower in patients who develop postoperative complications. CD19 lymphocytes were more present in complicated appendicitis.

## 1. Introduction

Appendicitis is a progressive, irreversible disease, and today, it is accepted in the literature that there are two different entities, simple (uncomplicated) and complicated appendicitis [1,2]. Despite the large number of described biochemical markers, use of different imaging methods, and scoring systems, the rates of late diagnosis and complications in children are still high [3]. Although acute appendicitis is one of the most common causes of abdominal surgical emergencies, until recently, the human appendix has been regarded as a rudimentary part of the intestine. The immunological function of the appendix as part of the gut-associated lymphoid tissue and its immunological importance for developing and preserving the intestinal immune system is not yet clearly understood [4,5]. The literature is scarce on immune mechanisms and cellular infiltrate composition in appendicitis, as only several studies have been conducted on the topic. Therefore, we sought some answers by analyzing the lymphocyte population in appendiceal specimens and inflammatory markers from peripheral blood. This study aimed to quantify different types of lymphocytes, CD8 T-lymphocytes, CD19, and CD20 B-lymphocytes, in simple and complicated appendicitis in children. Additional endpoints were to determine whether there is a difference in the cellular composition of appendicular infiltrate among different genders and age groups, and in patients who developed complications. It also sought to establish if there is a relationship between CD8, CD19, and CD20 lymphocytes in appendiceal specimens and hematological and biochemical parameters from peripheral blood.

## 2. Materials and Methods

### 2.1. Patients and Samples

The study included 100 patients aged 3–18 years, who were classified into two groups. The first group consisted of patients who underwent appendectomies due to acute appendicitis (N = 80), and the second group was the control group (N = 20 patients) who had appendectomies during some other abdominal surgery. Patients who underwent appendectomy in the control group had the following diagnoses: Intususception n = 6 (appendix was a part of intussusception), Meckel’s diverticulum n = 5, ovarian cyst n = 5, Amayand inguinal hernia n = 1, hernia of the umbilical cord n = 1, malrotation n = 1, and Hirschsprung’s disease n = 1. In those cases, the surgeon decided to perform an appendectomy due to some surgical concerns that could occur in time. Patients with chronical appendicitis and with hematological and immunological disorders were excluded from the study. The first group of patients consisted of two groups: Group A-patients with simple appendicitis (Acute and Suppurative appendicitis) and Group B-patients with a complicated form of appendicitis (Gangrenous and Perforated appendicitis). Peripheral blood samples were obtained preoperatively, and all anamnestic data were collected from the medical history. The follow-up period was one year after the last patient included in the study was operated. The research was conducted according to the Declaration of Helsinki (as revised in 2013) after Ethical Committee approval No: 14396/5 from 26.5.2022 was obtained, as was informed consent from all the patients or their families.

### 2.2. Tissue Sections, Monoclonal Antibodies, and Quantitative Analysis

Appendiceal specimens obtained at surgery were routinely prepared for histopathological examination. Histologic diagnosis was established by conventional hematoxylin-eosin staining (H&E) of the paraffin-embedded 3–4 μm thick sections. Two pathologists confirmed the histopathological diagnosis.

For immunohistochemical analysis of lymphocyte subtypes CD8, CD19, and CD20 in appendiceal specimens, the Ultravision LP-HRP polymer detection technique was used. A panel of commercially available mouse monoclonal antibodies (mAbs) was used to detect CD8, CD19, and CD20 lymphocytes. Primary monoclonal mouse anti-CD8, mouse anti-CD19, and mouse anti-CD20 antibodies were used (Leica Biosystems, Clone 4B11, Clone BT51E, and Clone L26, dilution 1:50, for 60 min at room temperature), and a secondary antibody (Vectastain Elite ABC kit, Vector Laboratories, Burlingame, CA, USA). Immune complexes were visualized by DAB (*Dako Liquid DAB + Substrate Chromogen System*, K3467) and counterstained with Mayer hematoxylin.

Semiquantitative analysis was used to quantify the presence of immunopositive cells in appendiceal specimens. The images for quantitative analysis were obtained on an Olympus BX50 light microscope equipped with a Leica DFC 295 digital camera (Leica Micro-System, Rueil-Malmaison, France). Photomicrographs taken at magnification ×200 were examined by digital image analysis software “ImageJ” version 1.43 (public domain software, Wayne Rasband, National Institutes of Health, Bethesda, MD, USA).

Statistical analysis: The data were analyzed statistically using IBM SPSS Statistics for Windows, Version 16.0.(IBM Corp., Armonk, NY, USA) The significance was defined as *p* < 0.05. The numerical variables between the two groups were compared using the t-test or the Mann–Whitney test. The Kruskal–Wallis test compared CD8, CD19 and CD20 between age categories. The Spearman correlation coefficient was used to estimate the association between the CD8, CD19, and CD20 lymphocytes and any hematological and biochemical parameters. Multivariate Cox regression analysis was used to estimate the association of complications in the follow-up period concerning selected demographic and clinical parameters.

## 3. Results

### 3.1. Results of Immunohistochemistry

**Figure 1:**
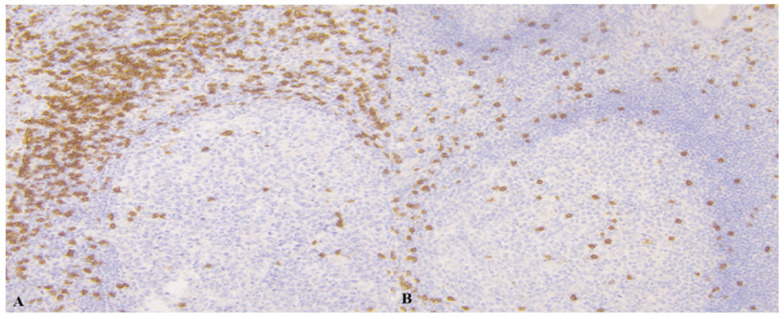
Photomicrographs of appendicitis specimens. Inflammatory cells infiltrated the appendiceal wall. An Immunostain of CD8 T-lymphocytes in (**A**) simple appendicitis and (**B**) complicated appendicitis; magnification 200.

**Figure 2:**
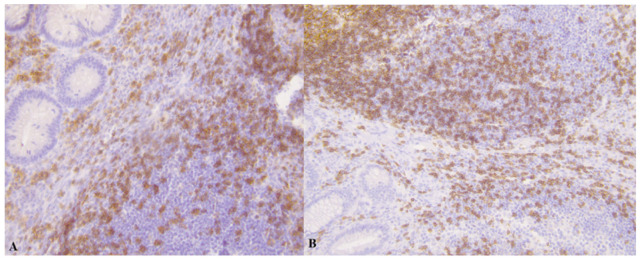
Photomicrographs of appendicitis specimens. Inflammatory cells infiltrated the appendiceal wall. An Immunostain of CD19 B-lymphocytes in (**A**) simple appendicitis and (**B**) complicated appendicitis; magnification 200.

**Figure 3:**
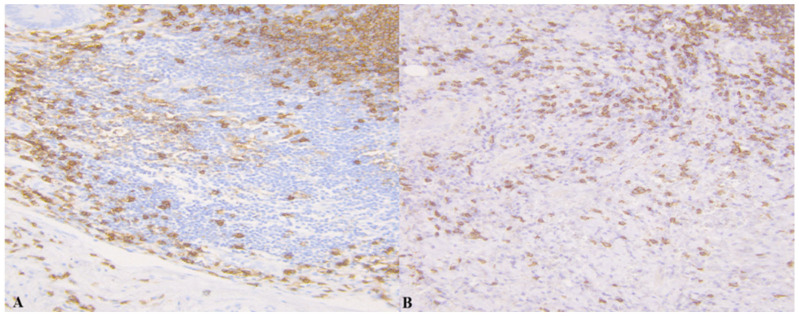
Photomicrographs of appendicitis specimens. Inflammatory cells infiltrated the appendiceal wall. An Immunostain of CD20 B-lymphocytes in (**A**) simple appendicitis and (**B**) complicated appendicitis; magnification 200.

**Figure 4:**
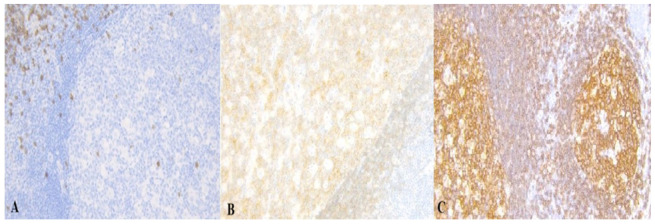
Photomicrographs of appendicitis specimens in the control group. Inflammatory cells infiltrated the appendiceal wall. An Immunostain (**A**) CD8, (**B**) CD 19, and (**C**) CD20 lymphociytes in the appendiceal wall; magnification 200.

In the following Figure 1, Figure 2, Figure 3 and Figure 4 there are illustrative photomicrographs representing immunohistochemistry of all lymphocyte subtypes CD8, CD19, and CD20, in different appendiceal specimens (simple and complicated appendicitis, and in the control group).

### 3.2. Results of Statistical Analysis

The study included a total number of 100 patients: n = 20 in the control group where the appendix was removed during some other abdominal surgical procedure, and n = 80 in the group of patients with acute appendicitis. The patients with appendicitis were divided into two minor groups: n = 35 patients with simple appendicitis in Group A, and n = 45 patients with complicated appendicitis in Group B. There were 65.0% of male patients, and the mean age at the time of surgery was 10.8 ± 4.0 years for patients with appendicitis, and the average age was 8.2 ± 6.5 years for the control group. Patients with appendicitis were significantly older compared to the control group (*p* = 0.023).

The presence of lymphocyte subtypes CD8, CD19, and CD20 was analyzed in the cellular infiltrate in appendiceal specimens of patients with appendicitis and in the control group. Interestingly, CD19 B-lymphocytes were significantly more present in the appendiceal specimens of patients with complicated appendicitis compared to simple appendicitis (*p* = 0.007), and on the other hand, there were significantly more CD8 T-lymphocytes in patients with simple appendicitis compared to patients with complicated appendicitis (*p* < 0.001). However, no significance was found in comparison to the control group. Results are shown in Table 1.

**Figure 5:**
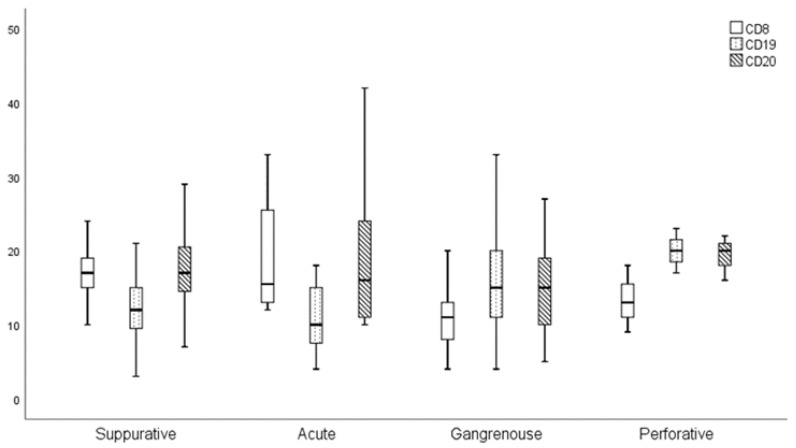
Presence of CD8, CD19, and CD20 in acute, suppurative, gangrenous, and perforative appendicitis.

As there are four different pathophysiological forms of appendicitis, the presence of CD8, CD19, and CD20 lymphocytes was evaluated and compared in all of them: acute, suppurative, gangrenous, and perforative appendicitis. CD8 lymphocytes were significantly more present in patients with acute appendicitis (*p* = 0.005). Results are shown in Figure 5.

There were no significant differences in the presence of CD8, CD19, and CD20 lymphocytes within age groups. (Table 2). Also, there was no statistically significant difference in lymphocyte subpopulations in appendiceal specimens regarding gender.

Hematological and biochemical parameters from peripheral blood were compared between Groups A, B, and the control group. Unlike the lymphocyte subtypes in the appendiceal specimens, here there is a statistically significant difference between the patients with appendicitis and the other from the control group. Significance is present in the following parameters linked with inflammation: Le (*p* < 0.001), Ne% (*p* < 0.001), count of Ne (*p* < 0.001), Ly% (*p* < 0.001), Ne/Ly ratio (*p* < 0.001), count of Mo (*p* = 0.048), Ne/Mo ratio (*p* = 0.001), and C-reactive protein (CRP) (*p* < 0.001). There was no significance between the groups of simple and complicated appendicitis. All results are presented in Table 3.

There is a statistically significant negative correlation in simple appendicitis between CD8 T-lymphocytes and the count of leukocytes (r = −0.342, *p* = 0.044). In a group of patients with complicated appendicitis, there is a statistically significant positive correlation between CD8 T-lymphocytes and the count of neutrophils (r = −0.342, *p* = 0.023) (Table 4).

Complications after appendectomy were present in 21.3% of patients. Early complications such as wound infections, sepsis, and SIRS (Systemic Inflammatory Response Syndrome) were present in 12.5%, and late complications like postoperative adhesive ileus and intra–abdominal abscess formation were present in 8.8% of patients. The average time from surgery to complication development was 80.44 ± 165.39 days, with a median of 12.50 days (Min 0 days, Max 638).

**Figure 6:**
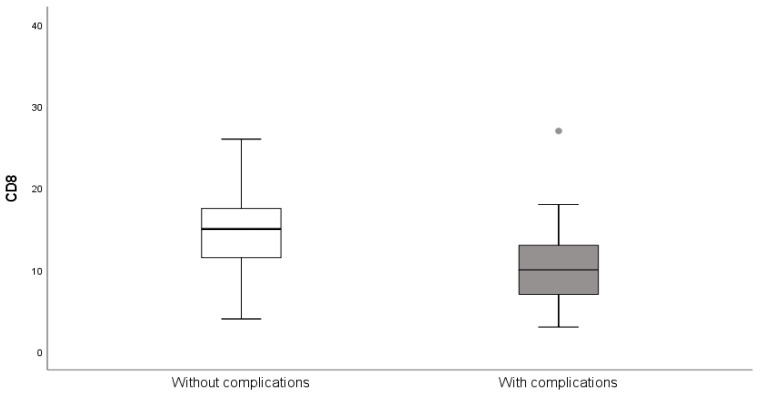
Presence of CD8 lymphocytes in patients with and without complications after appendectomy.

The patients who developed complications after appendectomies had significantly higher C-reactive protein (*p* = 0.040), and significantly fewer CD8 T-lymphocytes (*p* = 0.024) compared to patients without complications. However, no other hematological or biochemical parameter showed significance. Results are presented in Table 5 and Figure 6.

## 4. Discussion

In this research, the average age of patients with appendicitis at the time when appendectomy was performed was 10.8 ± 4.0 years, and there was a slight male predominance at 65.0%, although there was no statistical significance. This correlates with results in the studies conducted by Minneci et al. and Bansel et al., as they both reported that the highest incidence of appendicitis was in the second decade of life, and that it was more common in boys [6,7].

The literature is loaded with studies based on the role of different hematological and biochemical parameters as affordable tools in improving the accuracy of diagnosing appendicitis. For example, Saaiq et al. published that increased leukocytes can be a predictor for appendicitis with a sensitivity of 91.81%, specificity of 43.55%, positive predictive value of 81.77%, and negative predictive value of 65.85% [8]. The other study concluded that increased leukocytes have a greater sensitivity of 88% compared to C-reactive protein with 69%, while the lowest sensitivity of 60% was combined values of CRP and leukocytes [9]. Rodríguez–Sanjuán et al. proved that in children, there are increased levels of CRP in appendicitis, and levels of CRP are correlated with inflammation of the appendix [10]. All these findings correlate with the results of this study, as leukocytes and CRP were significantly increased compared to the control group with simple and complicated appendicitis.

As it is proven that the diameter of lymphatic follicles in the human appendix decreases with age, and lymphatic tissue is replaced with fibrosis while the muscular layer stays without changes, one of the additional endpoints of this research was to determine whether there is a difference in the cellular composition of appendiceal infiltrate within different age groups [11,12]. However, the findings of lymphocyte subtypes did not differ significantly in gender and age groups.

Analyzing the cellular infiltrate in the appendix of patients who underwent appendectomy, one lymphocyte subtype has been distinguished as more important than others. There was a significant predominance of cytotoxic CD8 T-lymphocytes in simple appendicitis than in complicated appendicitis. A similar study was conducted by Gorter et al., with the exception that the study did not have a control group. Gortner’s results correlate with the CD8 lymphocyte results of this research, as they found that there were fewer CD8, CD20, and CD21 in the complicated appendicitis group than in the simple appendicitis group. Also, the level of CRP was significantly higher in a group with complicated appendicitis. Gorter provided a potential explanation that a higher number of neutrophils and a decreased number of CD8, CD20, and CD21 in complicated appendicitis may trigger innate immune responses and reduce potential regulatory responses by adaptive immune cells [13]. Their results of the CD20 lymphocytes do not match with this study, as no significance was found. Tsuji et al. also found that CD8 and CD4 lymphocytes were present more in appendiceal specimens with acute focal and suppurative appendicitis than in the control group [14]. In the acute phase of appendicitis, the activation of CD8+ T cells is paramount. These cells produce pro-inflammatory cytokines such as tumor necrosis factor-alpha (TNF-α) and interferon-gamma (IFN-γ), essential for orchestrating the immune response against pathogens. The role of these cytokines is not only to enhance the cytotoxic activity of CD8+ T cells but also to recruit other immune cells to the site of infection [15]. Besides their cytotoxic functions, CD8+ T cells may also interact with other immune cell types, including CD4+ T helper cells, to modulate the overall immune response in appendicitis, which is critical for achieving a balanced immune response that effectively clears the infection while minimizing collateral damage to the host’s tissues [16]. On the contrary, Kuga et al. published that the greater predominance of CD8 T-lymphocytes and that natural killer cells were present in complicated appendicitis. In their study, the number of CD19 was higher in perforated appendicitis than in others, 70% vs. 63.2%, but without statistical significance, which correlates to the findings in this research. The limitation of Kuga’s research is that it was performed only on 27 patients, and unlike this study, the population was heterogeneous and consisted of children and adults [17].

Mosayebi et al.’s study has shown that CD19 B–lymphocytes have a significant role in the inflammatory response in acute appendicitis. They analyzed CD19 lymphocytes in appendiceal specimens and in peripheral blood and concluded that CD19 was more present in patients with appendicitis than in patients where appendicitis was not confirmed on pathohistological examination. Moreover, the level of CD19 in peripheral blood was reduced 48–72 h after appendectomy. They found that leukocytes, lymphocytes, and polymorphonuclears from peripheral blood and subpopulations of lymphocytes CD3, CD4, and CD8 were statistically insignificant, which is the opposite of the results of this study [4]. The relationship between CD19+ B cells and other immune cell types, such as helper T lymphocytes (CD4+) and cytotoxic T lymphocytes (CD8+), is also relevant. These interactions may enhance the overall immune response in the inflamed appendix [18].

A small sample study on 17 patients was performed by Somekh et al., and they published that CD19 B-lymphocytes were more present in peripheral blood and appendiceal specimens in patients with appendicitis than in those without appendicitis. They also discovered that expression of CD19 B-lymphocytes was reduced in specimens compared to peripheral blood. A possible explanation was that the human appendix is a place for diversification of B lymphocytes and that reduced expression is because CD19 lymphocytes in the appendix are part of GALT (Gut Associated Lymphoid Tissue), and they are functionally different [19]. The immunohistochemical analysis showed a greater number of CD20 B-lymphocytes, memory cells CD45R0, Ki-67+ cells, CD3, CD4, CD8, and CD45 T lymphocytes in the suppurative appendicitis compared to the control group, which differs from the results of this study [20].

The importance of lymphocytes in the pathophysiology of appendicitis is also documented in another study. Kang et al. have established machine learning models (Logistic regression) for preoperative prediction of the pathological types of acute appendicitis based on peripheral blood biomarkers and clinical symptoms. In their research, the number of natural killer cells decreased with the stage of inflammation; on the other hand, the number of CD19 increased with inflammation but without significance. The levels of CD8 lymphocytes and CRP were significantly higher in patients with complicated appendicitis [16]. It must be noted that the methodology of their study is different, as they performed their research solely from blood samples, and appendiceal specimens were not analyzed immunohistochemically; therefore, the results are not quite comparable with this research.

Complications of appendicitis include generalized peritonitis, sepsis, intra–abdominal abscesses, and ileus, and rates of complications are higher in patients with complicated appendicitis (up to 39%) compared to uncomplicated appendicitis (8%) [21,22]. The rate of complications for both complicated and simple appendicitis in this study was 21.3% which agrees with the literature data. Analysis of appendiceal specimens of patients with complications after appendectomy showed that CD8 T-lymphocytes were less present in patients with complications. One of the possible explanations is that the number of CD8 lymphocytes is also reduced in complicated appendicitis, which is more prone to complications. Unfortunately, it is impossible to compare results with other studies; as far as we know, no similar study has been performed yet.

## 5. Conclusions

Considering that this study was conducted on the largest number of solely pediatric patients, including the control group, it can be concluded that cytotoxic CD8 T-lymphocytes and CD19 B-lymphocytes play a significant role in the local immune response in acute appendicitis. Cytotoxic CD8 T-lymphocytes were more present in patients with simple appendicitis, and less present in complicated appendicitis and in patients who developed complications after appendectomy, which could have a predictive value in complication development. On the other hand, CD19 B-lymphocytes were significantly more present in the appendiceal specimens of patients with complicated appendicitis compared to those with simple appendicitis. However, no significant difference was found in the cellular composition of appendiceal infiltrates based on age and gender, and the presence of CD20 B-lymphocytes showed no significance at all.

## Figures and Tables

**Table 1:** Values of CD8, CD19, and CD20 in appendiceal specimens in Groups A, B, and the control group.

	Control Group	Group A	Group B	*p* ^1a^	*p* ^1b^
CD 8%	15.00 ± 4.60	16.83 ± 5.67	11.98 ± 5.65	0.417	<0.001
CD 19%	12.65 ± 3.73	12.14 ± 5.53	16.24 ± 7.68	0.407	0.007
CD 20%	20.30 ± 9.76	17.37 ± 7.21	16.09 ± 7.88	0.073	0.186

^1^ Mann–Whitney U test, ^a^ appendicitis vs. control group, ^b^ group A vs. group B, arithmetic mean ± standard deviation.

**Table 2:** Presence of CD8, CD19 and CD20 in different age groups.

†	<7 Years	8–13 Years	14+ Years	*p* ^1^
CD8%	12.95 ± 4.57	14.28 ± 5.96	14.86 ± 7.66	0.875
CD19%	14.65 ± 8.83	15.08 ± 6.64	13.1 ± 6.16	0.544
CD20%	17.4 ± 10.23	15.74 ± 5.79	17.62 ± 7.77	0.683

^1^ Kruskal–Wallis test, † arithmetic mean ± standard deviation.

**Table 3:** Hematological and biochemical parameters in groups.

Parameter ^†^	Control Group	Group A	Group B	*p* ^1a^	*p* ^1b^
Le	10.73 ± 5.71	17.86 ± 6.39	17.4 ± 5.44	<0.001	0.957
Ne%	59.16 ± 19.2	77.51 ± 8.13	78.89 ± 7.94	<0.001	0.426
Ne (count)	6.79 ± 5.05	16.05 ± 12.2	13.47 ± 5.06	<0.001	0.594
Ly%	29.89 ± 17.16	13.57 ± 6.74	12.71 ± 6.71	<0.001	0.541
Ly (count)	2.73 ± 1.8	2.19 ± 0.94	2.1 ± 1.13	0.236	0.447
Ne/Ly	3.99 ± 5.61	8.98 ± 8.33	8.5 ± 6.2	<0.001	0.945
Mo%	8.02 ± 3.79	6.38 ± 2.41	6.25 ± 2.64	0.061	0.828
Mo (count)	0.92 ± 0.74	1.1 ± 0.47	1.08 ± 0.51	0.048	0.917
Ne/Mo	9.67 ± 6.77	15.7 ± 10.48	15.53 ± 11.54	0.001	0.775
CRP	21.94 ± 33.94	66.75 ± 78.6	103.93 ± 85.04	<0.001	0.014
Na	128.53 ± 32.03	136.8 ± 2.65	135.97 ± 3.57	0.800	0.356
Albumin	38.38 ± 5.5	38.66 ± 4.2	38.83 ± 4.3	0.948	0.868
Glycemia	30.84 ± 105.19	5.48 ± 1.45	5.65 ± 1.39	0.575	0.246
Total bilirubin	31.22 ± 47.36	14.92 ± 12.67	17.43 ± 15.13	0.991	0.813
Direct bilirubin	3.23 ± 1.82	3.57 ± 2.18	7.67 ± 8.97	0.063	0.015

^1^ Mann–Whitney U test, ^a^ appendicitis vs. control group, ^b^ group A vs. group B ^†^ arithmetic mean ± standard deviation.

**Table 4:** Correlation of inflammatory markers between simple and complicated appendicitis.

		**Group A**				**Group B**			
		CD8	CD19	CD20	NK ćelije	CD8	CD19	CD20	NK ćelije
Le	r	−0.342 *	−0.078	−0.138	0.101	0.286	0.215	0.091	0.055
	*p*	0.044	0.654	0.428	0.564	0.057	0.157	0.553	0.721
	N	35	35	35	35	45	45	45	44
Ne%	r	−0.150	0.154	−0.111	−0.044	0.129	0.152	−0.032	−0.065
	*p*	0.389	0.376	0.527	0.803	0.398	0.319	0.837	0.677
	N	35	35	35	35	45	45	45	44
Ne (count)	r	−0.292	0.072	−0.075	0.132	0.342 *	0.248	0.006	0.064
	*p*	0.089	0.683	0.671	0.448	0.023	0.104	0.969	0.684
	N	35	35	35	35	44	44	44	43
Ly%	r	0.260	−0.072	0.087	0.026	−0.091	−0.114	−0.033	0.174
	*p*	0.132	0.679	0.621	0.884	0.557	0.463	0.832	0.265
	N	35	35	35	35	44	44	44	43
Ly (count)	r	0.044	−0.157	0.051	−0.020	0.140	−0.026	−0.042	0.259
	*p*	0.801	0.367	0.770	0.908	0.365	0.869	0.786	0.094
	N	35	35	35	35	44	44	44	43
Ne/Ly	r	−0.243	0.156	−0.051	0.017	0.077	0.098	−0.018	−0.172
	*p*	0.160	0.372	0.773	0.922	0.620	0.527	0.909	0.271
	N	35	35	35	35	44	44	44	43
Mo%	r	0.012	−0.209	0.316	0.059	−0.090	−0.159	0.036	−0.265
	*p*	0.947	0.229	0.064	0.737	0.563	0.303	0.817	0.085
	N	35	35	35	35	44	44	44	43
Mo (count)	r	−0.256	−0.193	0.180	0.140	0.126	0.046	0.080	−0.090
	*p*	0.137	0.267	0.301	0.424	0.414	0.764	0.605	0.568
	N	35	35	35	35	44	44	44	43
Ne/Mo	r	−0.085	0.319	−0.185	0.002	0.093	0.189	−0.040	0.214
	*p*	0.626	0.062	0.286	0.992	0.548	0.220	0.798	0.169
	N	35	35	35	35	44	44	44	43
CRP	r	−0.284	−0.161	0.021	0.075	0.020	−0.025	−0.073	−0.237
	*p*	0.098	0.357	0.903	0.668	0.898	0.871	0.635	0.122
	N	35	35	35	35	45	45	45	44

r—Spearman correlation rang coefficient.

**Table 5:** Presence of lymphocyte subtypes CD8, CD19, and CD20 in patients with and without complications after appendectomy.

†	Complications	Without Complications	*p* ^1^
CD8%	11.29 ± 6.16	14.86 ± 5.93	0.024
CD19%	16.06 ± 8.03	14.02 ± 6.82	0.403
CD20%	16.06 ± 10.68	16.81 ± 6.6	0.437

^1^ Mann–Whitney test, † arithmetic mean ± standard deviation.

## Data Availability

The datasets used and/or analyzed during the current study are available from the corresponding author on reasonable request.

## Abbreviations

WBCC	white blood cell count per μL of blood
ANC	absolute neutrophil count
ALC	absolute lymphocyte count
AMC	absolute monocyte count
ANC/ALC	neutrophil–to–lymphocyte ratio
ANC/AMC	neutrophil–to–monocyte ratio
CRP–C	reactive protein
